# Role of Obesity in Inflammation and Remodeling of Asthmatic Airway

**DOI:** 10.3390/life12070948

**Published:** 2022-06-23

**Authors:** Harshita Shailesh, Ibrahim A. Janahi

**Affiliations:** 1 Department of Medical Education, Sidra Medicine, Doha 26999, Qatar; hshailesh@sidra.org; 2 Department of Pediatric Medicine, Sidra Medicine, Doha 26999, Qatar; 3 Weill Cornell Medicine, Doha 24144, Qatar

**Keywords:** obesity, asthma, inflammation, airway remodeling, airway epithelial cells, airway smooth muscle cells, lung fibroblasts

## Abstract

Obesity is considered as an important risk factor for the onset of asthma and plays a key role in enhancing the disease’s severity. Obese asthmatic individuals represent a distinct phenotype of asthma that is associated with additional symptoms, more severe exacerbation, decreased response to standard medication, and poor quality of life. Obesity impairs the function of the lung airway in asthmatic individuals, leading to increased inflammation and severe remodeling of the bronchus; however, the molecular events that trigger such changes are not completely understood. In this manuscript, we review the current findings from studies that focused on understanding the role of obesity in modulating the functions of airway cells, including lung immune cells, epithelial cells, smooth muscle cells, and fibroblasts, leading to airway inflammation and remodeling. Finally, the review sheds light on the current knowledge of different therapeutic approaches for treating obese asthmatic individuals. Given the fact that the prevalence of asthma and obesity has been increasing rapidly in recent years, it is necessary to understand the molecular mechanisms that play a role in the disease pathophysiology of obese asthmatic individuals for developing novel therapies.

## 1. Introduction

Asthma is a major non-communicable disease that affects both the pediatric and adult populations worldwide. Asthma’s prevalence has been increasing significantly worldwide in recent years. A global study recently reported that approximately 300 million people are affected by asthma worldwide, and around 1000 people die from asthma every day [1]. Clinically, asthma is considered as a chronic lung disease associated with airway limitation due to a combination of pathophysiological events, including hypersensitivity, the inflammation and remodeling of the airway, and hypersecretion of the mucus leading to reduced airway function [2]. Obesity is considered as a major health problem. Extensive epidemiological studies have established a strong link between asthma and obesity, and indicated that obesity can serve as a major predisposing factor for asthma onset in children and adults (Table 1), [3,4,5,6]. A meta-analysis using different prospective pediatric cohort studies discovered a dose–response association between asthma and body weight, and revealed that overweightness and obesity increase the asthma risk by 20% and two-fold, respectively, in children [7]. As compared with all other asthma phenotypes, obese asthmatic patients are associated with additional symptoms, poor disease control, a higher rate of exacerbation, attenuated response to corticosteroid treatment, and reduced quality of life. Furthermore, most of the asthmatic patients that fall under the difficult-to-treat category are found to be obese [8,9].

Although most of the epidemiological studies have not addressed the causality of obesity-associated asthma, few studies have revealed that increased body mass index (BMI) percentile is associated with lower values of forced expiratory volume in 1 s (FEV1) and forced vital capacity (FVC), elevated levels of oxidative stress markers, and increased bronchial hyperresponsiveness [12,13,32,33,34,35,36,37]. A growing body of evidence is indicating that obesity-associated inflammation might also serve as a major trigger to asthma onset [38]. For example, an in vivo study by Calixto et al., 2010, showed that ovalbumin (OVA) exposure increases the eosinophil population in bone marrow and connective tissue in close proximity with the bronchial and bronchiolar segments of diet-induced obese C57BL/6 mice compared with that of the lean littermates. In addition, the bronchoalveolar lavage fluid (BALF) of obese mice had increased levels of inflammatory markers, including Interleukin (IL)-5, IL-10, eotaxin, and Tumor necrosis factor-α (TNF-α), as compared with those from their lean counterparts, highlighting the fact that obesity can promote the inflammation of the lung, a common clinical feature of asthma [23]. Similarly, mounting evidence from clinical studies further supports the fact that obesity increases lung inflammation in asthmatic patients [39,40].

Airway remodeling, an important pathophysiology of asthma, is regulated by different kinds of cells of the bronchial airway that include immune cells, epithelial cells, smooth muscle cells, and fibroblasts. Several recent investigations have indicated that obesity can alter the cellular landscape of the airway through different mechanisms, leading to its inflammation and remodeling (Table 1) [25,31,41,42,43]. In the current manuscript, we will summarize recent findings that explain the mechanisms through which obesity impacts airway cells, leading to airway remodeling and inflammation in asthma. The manuscript also highlights the opportunities for future research to understand the disease pathophysiology and develop novel therapeutic strategies to combat disease severity in obesity-associated asthma.

## 2. Obesity and Asthmatic Airway

**Figure 1:**
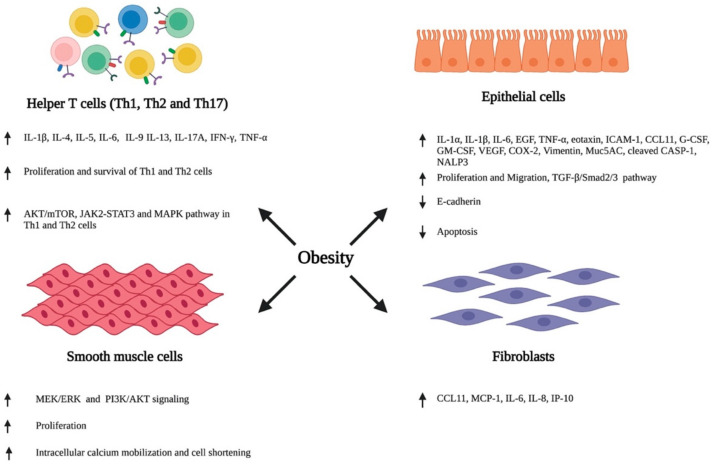
Obesity-associated alterations in the airway. Obesity induces the phenotypic switching of immune cells, epithelial cells, smooth muscle cells, and fibroblasts residing in the lung airway, consequently promoting their ability to secrete various inflammatory cytokines and chemokines, and altering their growth characteristics, including proliferation, migration, and apoptosis. ↑- increase; ↓- decrease. Abbreviation: IL: Interleukin; IFN-γ: Interferon-γ; TNF-α: Tumor necrotic factor- α, Th: T helper cell; EGF: Epidermal growth factor; ICAM-1: Intercellular adhesion molecule 1; CCL11: C-C Motif chemokine ligand 11; G-CSF: Granulocyte colony-stimulating factor; GM-CSF: Granulocyte macrophage colony-stimulating factor; VEGF: Vascular endothelial growth factor; COX2: Cyclooxygenase 2; Muc5AC: Mucin 5AC; CASP-1: Caspace 1; NALP3: Natch domain-, leucine-rich repeat-, and PYD-containing protein 3; TGF-β: Transforming growth factor-β; MCP-1: Monocyte chemoattractant protein-1; IP-10: Interferon gamma-induced protein 10.

Various external stimuli, including environmental allergens and infectious agents, trigger an inflammatory response in the bronchial airway through the secretion of various cytokines and chemokines by the structural and immune cells of the airway. This promotes the infiltration of circulating immune cells, resulting in an inflammatory insult in the airway. Furthermore, airway cells also change their cellular phenotype and differentiate, consequently resulting in a series of events, such as the hypersecretion of inflammatory cytokines, airway wall thickening, subepithelial fibrosis, increased neovascularization, and enhanced proliferation and hypertrophy of smooth muscle cells, leading to the impairment and obstruction of the airway [2,44,45]. Recent studies have reported that obesity plays a detrimental role in modulating the phenotypes of airway immune and other structural cells, consequently resulting in the inflammation, hyperresponsiveness, and structural remodeling of the airway (Figure 1).

### 2.1. Obesity-Associated Modulation of Lung Immune Cells

A diverse population of immune cells residing in the lung plays a major role in defense surveillance. The aberrant activation of lung immune cells leads to the increased inflammation and remodeling of the airway, a commonly observed phenomenon in asthma [46]. For instance, the activation of T helper cells (Th) residing in the lungs, such as Th2 and Th17, promotes the inflammation, mucous secretion, and remodeling of the airway in asthma via the secretion of different cytokines, such as IL-4, IL-5, IL-13, and IL-17, and interferon-γ (IFN-γ) [47]. Alveolar macrophages are another type of lung immune cell that help in host defense by inducing inflammation via phagocytosis and the release of apoptotic bodies. Lung macrophages polarize into either the M1 phenotype during a non-allergic trigger or the M2 phenotype during allergic sensitization [48]. The polarization of alveolar macrophages into the M2 phenotype is often observed in asthma [49,50]. The activated alveolar macrophages are associated with defective phagocytosis, efferocytosis, altered remodeling and repairing of the airway, and increased activation of intracellular inflammasomes, thus indicating their role in asthma pathogenesis [51]. In addition to these cells, lung immunity is also regulated by innate lymphoid cells (ILCs), a type of innate lymphocyte that synthesize various cytokines and promote inflammation and airway remodeling in asthma. For example, the induction of asthma phenotypes in mice by the administration of IL-25 or IL-33 through the intranasal route resulted in the accumulation of ILC2 in their BALF, lung, and mediastinal lymph nodes. Flow cytometry analysis showed that the accumulated ILC2 in lungs and BALF were associated with increased production of inflammatory cytokines, including IL-5 and IL-13. Similar outcomes were observed in house dust mite- or OVA-challenged asthmatic mice, implying that ILC2 plays a major role in inducing airway inflammation in asthma by enhancing the production of Th2 cytokines, such as IL-5 and IL-13 [52].

**Figure 2:**
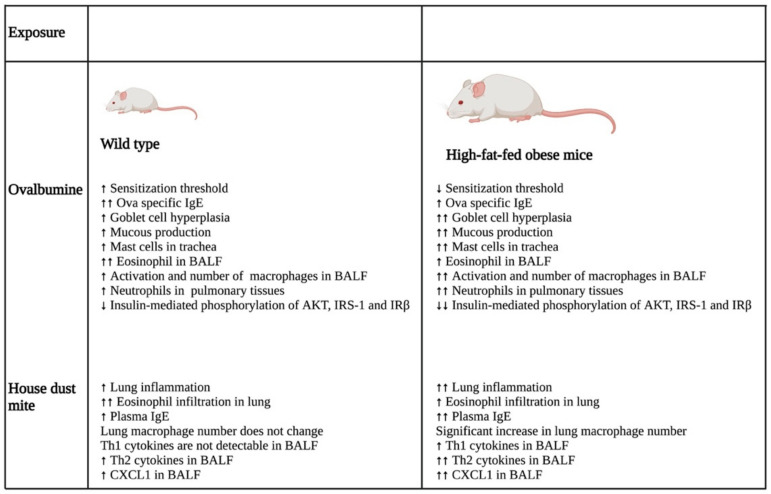
Differential impacts on lung inflammation and remodeling after allergen exposure in normal and high-fat-fed in vivo mice models. In vivo studies indicated that normal diet-fed- and high-fat-fed mice had significant differences in inflammation, with the latter being associated with increased lung inflammation and remodeling, enhanced cytokine levels, and an elevated number of immune cells. ↑- increase; ↑↑- further increase due to additional effect; ↓- decrease; ↓↓- further decrease due to additional effect. Abbreviations: IgE: Immunoglobulin E; BALF: Bronchoalveolar lavage fluid; IRS-1: Insulin receptor substrate-1; IRβ: Insulin receptor β; CXCL1: Chemokine (C-X-C motif) ligand 1.

A study that investigated the impact of obesity on allergic inflammation in the airway indicated that obesity has an important role in modulating the immune response of T helper cells and macrophages (Figure 2). OVA exposure results in increased expression of Th2 cytokines (IL-4, IL-5, IL-9, and IL-13) and Th17 cytokine, IL-17 A, in normal-weight BALB/c mice after 24 h, which was reduced after 48 h. On the other hand, high-fat-fed obese mice only had substantial increases in IL-9 and IL-13 after 24 h, which remained high even after 48 h of OVA challenge. Furthermore, these obese mice showed significant elevation of cytokines, such as IFN-γ, IL-4, IL-17A, TNF-α, IL-1β, and IL-6, after 48 h of OVA sensitization [28].

In the same study, the authors also showed that the OVA challenge resulted in the significant hyperplasia of goblet cells with increased mucous production in obese mice as compared with their normal-weight littermates. Furthermore, there was a substantial increase in the neutrophil population in the bone marrow of the obese group as compared with that of the lean group after 48 h of OVA sensitization. In addition, BALF analysis showed that the obese mice had a greater influx of macrophages in their BALF as compared with the lean mice after 24 and 48 h of OVA exposure. In contrast, eosinophil influx was lowered drastically in the BALF of obese mice as compared with that of the lean group at both time points. Furthermore, the macrophages in the allergic BALF of obese mice showed increased populations of arginase and inducible nitric oxide synthase-expressing macrophages as compared with those of non-obese, lean mice, indicating enhanced the activation of alveolar macrophages in the obese mice during allergic exposure. The pulmonary tissues of the obese mice had an increased number of neutrophils as compared with the lean mice after 48 h of OVA exposure. Collectively, the findings of the study imply that obesity impacts OVA-induced allergic inflammation in the airway of BALB/c mice by prolonging the inflammatory response of different Th cells, augmenting the influx of mixed granulocytes, and elevating macrophage activation and mucous production [28].

Another study investigated the role of obesity-associated adipokine, leptin, in activating lung immune cells and inducing airway inflammation in asthma using Ob^−/−^ leptin-deficient mice [27]. The study revealed that Ob^−/−^ mice showed reduced infiltration of lymphocytes and eosinophils in the BALFs and peribronchovascular spaces of their lungs compared with the wild-type littermates after the induction of experimental asthma by papain. Furthermore, inducing asthma by challenging with both papain and OVA resulted in reduced production of immunoglobulin (Ig) Es that are specific to OVA in the BALF and serum of Ob^−/−^ mice as compared with the control group, further indicating that leptin plays an essential role in inducing allergic airway inflammation in mice. Flow cytometry and enzyme-linked immunosorbent assay (ELISA) studies indicated that Ob^−/−^ asthmatic mice were associated with a reduced number of Th2 and ILC2 cells and decreased amounts of Th2 cytokines, including IL-4, IL-5, and IL-13, in their lung draining mediastinal lymph nodes as compared with the normal-weight asthmatic group. In complement to this finding, an in vitro study also indicated that exogenous leptin exposure promotes the expression of IL-4, IL-5, and IL-13 in Th2 cells without inducing their differentiation. Furthermore, leptin-deficient Ob^−/−^ asthmatic mice were also associated with fewer proliferating Th2 and ILC2 as compared with their normal-weight asthmatic littermates after the experimental induction of allergic asthma. In support of this, an in vitro study showed that leptin administration increases the proliferation of activated Th2 cells and promotes their survival. Since flow cytometry analysis showed the presence of leptin receptors on the surface of Th2 cells, the authors investigated the mechanism through which leptin induces their proliferation. Using specific inhibitors, the authors systematically demonstrated that leptin promotes the proliferation, survival, and cytokine production of Th2 cells by activating the PI3K-AKT-mTOR, JAK2-STAT3, and MAPK pathways [27]. Collectively, the study indicated that the obesity-associated hormone leptin plays an indispensable role in promoting the inflammation of allergic airways in asthma by activating lung immune cells and modulating the influx of circulating immune cells.

### 2.2. Role of Obesity in Altering Airway Epithelium

The airway epithelium serves as a frontline barrier between the environment and the host system, and is exposed to infectious agents and airborne particles [53]. Structurally, the epithelial layer is composed of ciliated columnar epithelial cells, intermediate columnal epithelial cells, goblet cells, basal cells, serous cells, and side population cells [54]. The epithelial cells of the airway are endowed with different pattern recognition receptors, including Toll-like receptors, which are activated upon allergen and infectious agent exposure in asthmatic individuals. The activation of these receptors stimulates the secretion of various chemokines and cytokines from epithelial cells, such as IL-6, IL-8, IL-12, IL-12p40, IL-25, IL-33, CCL(C-C Motif chemokine ligand) 2, CCL20, GMCF (Granulocyte macrophage colony-stimulating factor), serum amyloid-A, and TSLP (Thymic stromal lymphopoietin), which subsequently triggers dendritic cell recruitment to the epithelial membrane [55,56,57,58]. Recruited dendritic cells trigger the activation of innate and adaptive immunity, resulting in an inflammatory insult in the airway [59]. Airway epithelial cells also release IL-33 that activates Th2 cells to secrete IL-5 and IL-13. These cytokines of the interleukin family promote the eosinophilic inflammation and metaplasia of goblet cells, resulting in airway inflammation and hyperresponsiveness [60]. In addition, the airway epithelium also differentiates into a proliferative phenotype in asthma, leading to epithelial layer thickening in the bronchus [61]. Activated airway epithelial cells can also augment airway remodeling by promoting the migration of airway smooth muscle (ASM) cells into the epithelial layer. For instance, an in vitro study showed that epithelial cells infected with human rhinovirus secrete various chemokines, such as CCL5, CXCL (Chemokine (C-X-C motif) ligand) 8, and CXCL10. Among these secreted chemokines, only CCL5 promoted the migration of ASMs [62]. Taken together, these findings indicate that the epithelial cells of the airway play a pivotal role in inducing the inflammation and remodeling of the lung airway in asthma.

Recent studies have indicated that obesity induces the remodeling of airway epithelium via different mechanisms. A study by Elliot et al., 2019, showed that obese individuals are associated with excessive deposition of adipose tissue in the outer epithelial walls of medium and large airways that leads to airway wall thickening, consequently narrowing the airway [63]. Another study by Suzukawa et al., 2015, investigated the molecular mechanism through which obesity modulates airway epithelial cell functioning. They found that the obesity hormone leptin increases the expression of the cell adhesion molecule, intercellular adhesion molecule (ICAM-1), in the nuclear factor kappa light-chain enhancer of activated B cells (NF-κB) in a dependent manner in human-derived primary bronchial epithelial cells and the BASB-2B airway epithelial cell line in vitro [25]. ICAM-1 is a transmembrane glycoprotein that promotes the infiltration of eosinophils and neutrophils into the airway during inflammation [64,65]. The study also showed that the leptin-mediated upregulation of ICAM-1 in BASB-2B cells was abrogated by dexamethasone treatment, indicating that BASB-2B cells are responsive to steroid treatment after leptin exposure. Using the multiplex cytokine analysis assay, the same study showed that leptin stimulation also increases the secretion of different cytokines, including IL-6, CCL11, Granulocyte-colony stimulating factor (G-CSF), and vascular endothelial growth factor (VEGF), in BSAB-2B cells. Flow cytometry analysis indicated that BEAS-2B cells express the leptin receptor Ob-R. Furthermore, transfecting these cells with Ob-R siRNA, followed by leptin treatment, did not show any upregulation of CCL11 mRNA, confirming that leptin modulates the phenotype of BEAS-2B cells by directly binding to its receptor. The study also demonstrated that leptin increased the proliferation and migration of airway epithelial cells and was able to protect TNF-α and IFN-γ-induced cell apoptosis in these cells [25]. Taken together, the study indicated that leptin plays a key role in inducing the pro-inflammatory phenotype in airway epithelial cells. However, future investigations using in vivo models are necessary to confirm the role of leptin in inducing lung epithelial cell phenotypic switching in obesity-associated asthma.

Cysteinyl leukotrienes, a group of inflammatory lipid mediators, are found to be elevated in obese asthmatic individuals [66]. A study by Dholia and coworkers investigated the role of leukotriene D4 (LTD4) in promoting the inflammation and remodeling of airway epithelial cells in vitro [43]. LTD4 treatment increased the expression of inflammatory cytokines, such as IL-1α, IL-1β, IL-6, epidermal growth factor (EGF), TNF-α, granulocyte macrophage colony-stimulating factor (GM-CSF), and eotaxin, in small-airway epithelial cells (SAECs) in a time- and dose-dependent manner. The study also showed that LTD4 treatment induces the activation of the Natch domain-, leucine-rich repeat-, and PYD-containing protein 3 (NALP3) inflammasome by activating Caspase-1 and secreting IL-1β, indicating that LTD4 might serve as a danger molecule that binds to pattern recognition receptors on SAECs to induce inflammation. In support of this finding, LTD4 treatment also showed increased cyclooxygenase-2 (COX-2) inflammatory pathway activation in SAECs by enhancing the levels of COX-2 protein. In addition, using the air–liquid interface (ALI) technique, the authors of the study showed that LTD4 promotes the remodeling of SAECs by increasing the expression of vimentin and lowering the expression of E-cadherin, indicating the epithelial to mesenchymal transition of these cells. Furthermore, LTD4 treatment also increased the expression of Mucin5AC (Muc5AC), a marker of goblet cell hyperplasia, in SAECs grown in ALI culture. These molecular changes were associated with structural alterations in SAECs, such as the loss of cilia and excessive accumulation of mucin. Further investigation by the same group to understand the molecular mechanism involved in LTD4-mediated remodeling of SAECs indicated that LTD4 treatment increases the expression of transforming growth factor (TGF)-β that consequently phosphorylates Smad2/3 and activates the TGF-β/Smad2/3 pathway [43]. Collectively, the findings of the study indicate that an elevated level of LTD4 might serve as a trigger for airway remodeling in obese asthmatic individuals by altering the phenotype of small-airway epithelial cells.

### 2.3. Obesity-Induced Alteration of Airway Smooth Muscle Cell Phenotype

Airway smooth muscle cells form another important structural component of the airway that plays an important role in regulating the structure, function, and bronchomotor tone of the bronchial airway. In asthma, the smooth muscle cells of the bronchus induce the contraction of the airway by altering the intracellular Ca^2+^ levels [67]. Furthermore, the ASMs of asthmatic individuals show an increased size (hypertrophy), elevated proliferation rate (hyperplasia), and enhanced migration to the airway epithelial layer, leading to the increased thickness of the airway in asthma [68]. The thickness of the ASM area has been found to be positively linked to asthma severity independent of the disease period, indicating that ASM layer thickening is an early event in asthma that determines the severity of the disease [69]. Airway smooth muscle cells can also induce a proinflammatory environment in asthma by secreting a myriad of inflammatory cytokines, including a family of interleukins (IL), such as IL-1, IL-5, IL-6, and IL-8, and growth factors, including TGF-β1 and VEGF [70,71]. Thus, differentiated airway smooth muscle cells play a critical role in the inflammation, narrowing, and remodeling of the bronchial airway in asthma.

The increased activation of airway smooth muscle cells in response to various external and internal cues results in their hyper contraction by enhancing the release of calcium from the sarcoplasmic reticulum and subsequent phosphorylation of the myosin light chain. These events consequently lead to airway hyperresponsiveness in asthma [72,73,74]. Obesity is shown to aggravate airway hyperresponsiveness in asthmatic individuals to a higher extent. A study by Orfanos et al., 2018, investigated the impact of obesity on the airway smooth muscle cell response to contractile antagonists, carbachol, and histamine [30]. The results of their study showed that carbachol stimulation induces the enhanced phosphorylation of the myosin light chain in the human airway smooth muscle (HASM) cells of obese individuals as compared with those from age- and sex-matched lean individuals. Using single-cell calcium analysis, the authors of the study informed that both carbachol and histamine provoke intracellular calcium mobilization in HASM cell lines derived from obese subjects to a significantly higher extent than those originating from normal-weight donors. Among obese individuals, the HASM cells of women had higher intracellular calcium release in response to carbachol as compared with those from men. The HASM cells of obese females also showed increased intracellular calcium release as compared with those from comparable nonobese female counterparts; however, no significant difference was observed between the HASM cells of obese and nonobese males. Of note, the intracellular calcium response of HASM cells derived from obese donors was comparable to that of those obtained from fatal asthma patients, indicating that obese individuals can develop airway hyperresponsiveness to an extent similar to asthma patients. In support of these findings, a fluorescently labeled elastomeric contractible surfaces (FLECS) assay showed that the HASM cells of patients with obesity undergo greater shortening after carbachol and histamine exposure as compared with those of nonobese individuals. Taken together, the findings of this study indicate that obesity might play a dominant role in augmenting airway hyperresponsiveness in asthma by modulating the mechanism of cell contraction in airway smooth muscle cells [30].

Increased levels of free fatty acids (FFAs) are frequently reported in obese individuals [75]. Elevated levels of FFAs are known to induce the phenotypic switching of various types of cells, such as airway smooth muscle cells, by binding to their endogenous receptors on the cells’ surface. A systematic study by Matoba et al., 2017, showed that long-chain fatty acids, such as oleic acid and linoleic acid, and GW9508, an agonist of FFA receptor 1 (FFAR1), induce the proliferation of HASM cells in vitro by activating the MEK/ERK and PI3K/AKT pathways [76]. Mechanistically, long-chain FFAs and GW9508 activate MEK/ERK and PI3K/AKT signaling cascades by phosphorylating ERK and AKT in vitro in HSAM cell lines and patient-derived HASM cells, as well as in airway smooth muscle isolated from rats ex vivo. Further investigations from the same group also indicated that one of the long-chain FFAs, oleic acid, induces the phosphorylation of ERK and AKT in HASM cells by binding to the FFAR1 receptor that associates with G proteins, Gα_i_ and Gα_q_, on the cell membrane, leading to the dissociation of Gβγ subunits from Gα proteins. Furthermore, the study showed that FFAR1-coupled Gα_i_ and Gα_q_, and dissociated Gβγ subunits activate the c-Raf/ERK pathway, leading to ERK phosphorylation, whereas Gβγ_i_ induces the phosphorylation of AKT by activating ras and Src without involving Gβγ subunits. The study also revealed that the phosphorylation of AKT and ERK proceeds through PI3K and MEK, respectively, further leading to the phosphorylation of p70S6K, which eventually phosphorylates the S6 ribosomal protein. These events consequently promote airway smooth muscle cell proliferation. Collectively, the findings of this study indicate that elevated free fatty acids in obesity can play a key role in inducing the hyperplasia of airway smooth muscle cells, a key feature of asthma, by activating different proliferative signaling cascades [76].

### 2.4. Obesity and Airway Fibroblasts

Lung fibroblasts play a vital role in asthma pathogenesis by promoting airway remodeling and fibrosis by secreting different inflammatory cytokines and extracellular matrix proteins [77,78]. A recent investigation indicated that leptin exposure enhances the production of various cytokines and chemokines, such as eotaxin, monocyte chemoattractant protein-1 (MCP-1), IL-6, IL-8, and interferon gamma-induced protein 10 (IP-10), in normal lung fibroblasts. However, the siRNA-mediated knockdown of Ob-R receptors in these cells suppresses the leptin-mediated upregulation of IL-6, CCL11, and IP-10, indicating that leptin can play a detrimental role in promoting inflammation in asthma patients by stimulating fibroblast differentiation [31]. However, further investigations are needed to understand the other mechanisms through which obesity promotes fibrosis in asthma.

## 3. Potential Therapies

A significant number of clinical studies have shown that obese asthmatic individuals do not respond well to standard asthma therapy. An early study using a large cohort of asthmatic patients indicated that obese–asthmatic patients with BMI ≥ 40 kg/m^2^ were associated with reduced asthma control as compared with the non-obese group when treated with a combination of inhaled corticosteroid, fluticasone propionate, and long-acting β2 agonist (LABA), salmeterol; however, the study did not investigate the cause of the differential response between the two groups [79]. Some pharmacological interventions were also found to exacerbate asthma symptoms in obese asthmatic individuals. For example, a randomized clinical study indicated that obese asthmatics showed increased asthma symptoms when treated with theophylline, a phosphodiesterase inhibitor, as compared with a placebo group. In contrast, theophylline treatment reduced the asthma exacerbation rate in normal weight and overweight asthma patients [80]. Collectively, these findings indicate that careful tailoring is necessary when treating obese asthmatic patients using pharmacological agents.

**Figure 3:**
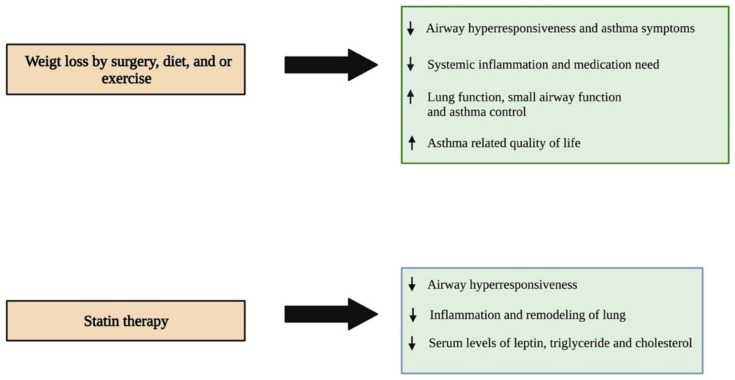
Different therapeutic approaches and their impacts on improving asthma symptoms in obese individuals. Reducing weight through different interventions has been shown to have many beneficial roles in improving asthma symptoms in obese individuals. Statin therapy is a novel pharmacological intervention that is showing a promising impact in treating asthma patients associated with obesity. ↑- increase; ↓- decrease.

Several studies in small cohorts have shown that weight loss by surgical intervention, diet, or exercise is found to be effective in improving lung functions and improving asthma symptoms (Figure 3). An early prospective study by Boulet et al., 2011, investigated the impact of weight loss followed by bariatric surgery in obese asthmatic individuals. The study results indicated that obese asthmatics showed a significant reduction in airway responsiveness, asthma symptoms, systemic inflammation, and medication need, with a noticeable improvement in lung function after twelve months of surgery. Of note, these changes were not observed in the control group of obese asthmatic individuals that did not undergo surgery [81]. Similar results were observed in another study which reported that bariatric surgery improved small airway function, asthma control, and lung function, and reduced airway hyperresponsiveness and systemic inflammation one year after the surgery in obese asthmatic patients. In addition, these individuals were associated with reduced need for asthma medication and increased daily physical activity [82]. Another recent study assessed the impact of weight loss on adipokine levels and lung function in obese asthmatic individuals that participated in a pre-operative weight loss program followed by bariatric surgery. Twelve months after surgery, the individuals showed a substantial reduction in the levels of IL-8, c-reactive protein, TNF-α, and leptin in their blood, and TNF-α in their induced sputum. However, the levels of IL-6 and adiponectin increased significantly in their blood. The study also showed that these patients were associated with improved asthma control. However, no improvement in pulmonary function was observed post-intervention [83].

Another prospective study by Pakhale et al., 2015, showed that diet-induced weight loss reduces airway hyperresponsiveness and improves lung function, asthma control, and asthma-related quality of life in obese asthmatic individuals as compared with a control group that received no diet intervention [84]. Another study by Freitas et al., 2018, showed that hypocaloric-diet-mediated weight loss in combination with increased daily life physical activity by aerobic and resistance exercise reduced asthma symptoms significantly in obese individuals with asthma as compared with a control group of obese asthmatics subjected to a hypocaloric diet and sham exercise, such as stretching and breathing exercises. In this study, obese asthmatics that underwent weight loss and exercise intervention showed an increase in the number of asthma-symptom-free days without coughing, shortness of breath, wheezing, nocturnal awakening, and the use of relief medications three months after the intervention when compared to the month before the intervention. On the other hand, the increase in the number of asthma-symptom-free days after three months of the intervention was considerably lower in the control group as compared with the experimental group [85]. Although these findings collectively indicate that weight loss and exercise serve as potential therapeutic approaches to reduce asthma symptoms that are worsened by increased body mass in obesity-associated asthma patients, further studies are needed using large cohorts to validate these findings.

Recently, researchers have started focusing on developing novel therapies for treating the obese asthmatic phenotype. Experimental studies have indicated that statin therapy is promising in the treatment of obesity-associated asthma (Figure 3). A study by Han and co-workers investigated the impact of a statin, simvastatin, in reducing the leptin level and improving dyslipidemia in a high-fat-diet-induced and OVA-exposed obese asthmatic mouse model [86]. The results of their study showed that obese asthmatic mice that received simvastatin or a combination of simvastatin and dexamethasone had reduced serum levels of leptin, triglyceride, and total cholesterol as compared with control obese asthmatic littermates that did not receive any treatment. In stark contrast, treatment with dexamethasone increased the levels of these serum biomarkers in the obese asthmatic mice as compared with the control group. BALF analysis showed that simvastatin alone or in combination with dexamethasone significantly reduces the eosinophil and neutrophil counts; however, dexamethasone alone could reduce only the eosinophil population in the obese asthmatic mice compared with the control group. The histopathological analysis of lung tissues indicated that the control obese asthmatic mice were associated with enhanced infiltration of eosinophils into perivascular and peribronchiolar connective tissues, increased the thickening of the airway, hyperplasia of goblet cells, increased mucous secretion, and caused noticeable collagen deposition as compared with the normal-weight asthmatic littermates. Interestingly, all three treatments significantly reduced eosinophil infiltration into the lungs, whereas only simvastatin and the combination treatment decreased collagen deposition, indicating that both simvastatin and dexamethasone have an anti-inflammatory effect, and only simvastatin can reduce airway remodeling in asthmatic mice with obesity [86]. Similar results were observed in another study that investigated the impact of pravastatin treatment in reducing airway inflammation in an obese asthmatic mouse model [87]. The administration of pravastatin through the intraperitoneal route reduced OVA-induced airway hyperreactivity in both lean and high-fat-fed obese mice. However, only obese OVA mice showed a significant reduction in inflammatory cells, including macrophages, eosinophils, and neutrophils, in their lung tissues. Furthermore, structural and cellular alterations, including increased bronchial epithelial layer thickening and infiltration of eosinophils and neutrophils into the peribronchial space after the OVA challenge, were effectively reversed by pravastatin in obese mice. However, lean OVA mice showed a reduction only in the epithelial layer of the lung, without having a significant change in the eosinophil and neutrophilic number after pravastatin treatment. Similarly, pravastatin treatment reduced the levels of cytokines, including IL-4, 5, and 17, in the BALF of the OVA-challenged obese mice, which was not observed in the lean OVA group. Pravastatin was also able to reduce the serum leptin levels and consequently MAPK pathway in the lung of obese OVA mice [87]. Collectively, these studies suggest that statins may serve as a potential therapeutic drug that can be used to reduce the serum levels of leptin and lipids, including triglycerides and total cholesterols, as well as airway inflammation and remodeling in obese asthmatic patients in the future.

## 4. Conclusions

Although asthma patients show some common clinical features, careful investigations by clinical researchers and molecular biologists have indicated that the disease has great heterogeneity in its clinical presentation and molecular mechanisms, resulting in distinct phenotypes and endotypes, respectively [2]. Obese asthmatic individuals represent a unique phenotype with more serious clinical complications of asthma, such as severe impairment in lung function, more frequent exacerbation, poor disease control, and reduced response to conventional therapies as compared with normal-weight asthma patients, causing increased health care expenditure [88,89]. The underlying mechanisms that play roles in inducing these manifestations in obese asthmatic individuals have not yet been studied extensively. However, some studies have indicated that obesity can promote the onset of asthma and enhance its severity through different mechanisms, which include the stimulation of low-grade systemic inflammation, alteration in metabolism, and modification of lung dynamics. Recently, few investigations have demonstrated that obesity-associated factors can activate lung cells, including Th cells, epithelial cells, smooth muscle cells, and fibroblasts, and promote them to secrete various cytokines and chemokines, consequently leading to the inflammation and remodeling of the airway. Increased inflammation and severe remodeling of the asthmatic airway in obese individuals serve as major risk factors for disease severity and a reduced response to conventional asthma therapies in these individuals. Therefore, it becomes important to conduct further investigations to delineate the molecular mechanisms underlying the obesity-associated inflammation and remodeling of the airway in asthma. Recognizing novel molecular events will eventually help to shift the paradigm of conventional therapy to more targeted treatments for obese asthmatic individuals, resulting in better outcomes.

## Figures and Tables

**Table 1:** Major clinical and basic research studies highlighting the association between obesity and asthma.

Author	Major Finding
	Clinical Research
Shaheen et al., 1999 [4]	Higher BMI during adulthood is significantly associated with increased asthma prevalence
Rönmark et al., 2005 [6]	Obesity (BMI ≥ 30) and overweightness (BMI 25.0–29.9) increase asthma risk by 2.7- and 2.0-fold, respectively, as compared with normal-weight individuals
Jones et al., 2006 [10]	Lung volumes, including functional residual capacity (FRC) and expiratory reserve volume (ERV), reduce exponentially with increasing BMI
Sood et al., 2006 [11]	Serum leptin level and BMI are positively associated with asthma in women
Komakula et al., 2007 [12]	BMI is associated with lower exhaled nitric oxide and higher levels of oxidative stress markers, 8-isoprostanes, in exhaled breath
Thyagarajan et al., 2008 [13]	Increase in BMI is associated with reduced lung function by lowering FEV1 and FVC
Ciprandi et al., 2009 [14]	Increased BMI is significantly associated with enhanced bronchial hyperresponsiveness in asthma patients
Dixon et al., 2011 [15]	Bariatric surgery has a beneficial impact on improving airway hyperresponsiveness in non-atopic obese asthmatic individuals
Schatz et al., 2013 [16]	Increased BMI is associated with enhanced seasonal asthma exacerbation risk in pediatric and adult populations having persistent asthma
Sanchez Jimenze et al., 2014 [17]	Insulin resistance increases the risk of allergic asthma in obese children and adolescents. Increased waist circumference is associated with reduced FVC and FEV1
Chen et al., 2017 [18]	Asthmatic children show higher risk (51%) of obesity during their later childhood and adolescence when compared with non-asthmatic children
To et al., 2018 [19]	Obesity is independently associated with reduced asthma control and increased exacerbation in severely asthmatic adult females
Luthe et al., 2018 [20]	Obese asthmatic individuals have increased acute severity of asthma that is associated with the enhanced use of mechanical ventilation and longer periods of hospitalization as compared with lean asthmatic individuals
Saheb Sharif Askari et al., 2019 [21]	Obese asthmatic children with BMI ≥ 85% are associated with increased asthma severity and enhanced frequency of hospital visits due to asthma as compared with lean individuals
Michalovich et al., 2019 [22]	Both obesity and asthma contribute additively to enhancing inflammation and microbiota alternation
	**Basic Research**
Calixto et al., 2010 [23]	High-fat-diet-induced obesity is associated with increased eosinophil migration from the bone marrow into lung tissue and enhanced expression of Th1 and Th2 cytokines, resulting in a prolonged stay of eosinophils in peribronchiolar segments of lungs
Dietze et al., 2012 [24]	HFD-induced obesity reduces the allergic sensitization threshold and increases eosinophilic airway inflammation in mice
Suzukawa et al., 2015 [25]	Obesity-associated hormone, leptin, promotes pro-the inflammatory phenotype, proliferation, migration, and apoptosis of airway epithelial cells
Diaz et al., 2015 [26]	Obese mice show reduced adiponectin level in plasma, decreased and increased infiltration of eosinophil and macrophages, respectively, into the lungs and BAL, increased expression of markers of macrophages (M1 and M2) in the lungs, and elevated expression of cytokines (Th1 and Th2) in BAL, and reduced response to dexamethasone as compared with lean mice upon house mite dust exposure
Zheng et al., 2016 [27]	Leptin augments inflammation in allergic asthma by activating lung immune cells
Silva et al., 2017 [28]	Obesity augments OVA-induced allergic inflammation in mice by prolonging the immune response by Th cells and increasing mixed granulocyte influx, macrophage activation, and mucous production
Andre et al., 2017 [29]	HFD-fed obese–asthmatic mice show impaired insulin signaling in their lungs due to reduced phosphorylation and enhanced tyrosine nitration of AKT, insulin receptor β, and insulin receptor substrate-1 as compared with lean-asthmatic mice
Orfanos et al., 2018 [30]	Airway smooth muscle cells of obese individuals show increased hyperresponsiveness to contractile antagonists as compared with those of lean individuals
Watanabe et al., 2019 [31]	Leptin induces the differentiation of lung fibroblasts by increasing the production of different inflammatory chemokines and cytokines

Abbreviations: BMI: Body mass index; FEV1: Forced expiratory volume in 1 s; FVC: Forced vital capacity; Th: T Helper cell; HFD: High-fat diet; BAL; Bronchoalveolar lavage; OVA: Ovalbumin.

## Data Availability

Not applicable.
